# Mobility and Aging: Transference to Transportation

**DOI:** 10.4061/2011/392751

**Published:** 2011-08-15

**Authors:** Thomas R. Prohaska, Lynda A. Anderson, Steven P. Hooker, Susan L. Hughes, Basia Belza

**Affiliations:** ^1^Institute for Health Research and Policy (MC 275), University of Illinois at Chicago, 1747 West Roosevelt Road, Chicago, IL 60608, USA; ^2^Healthy Aging Program, Centers for Disease Control and Prevention, 4770 Buford Highway, Atlanta, GA 30341, USA; ^3^Public Health Research Center, Arnold School of Public Health, University of South Carolina, Room 117, 921 Assembly Street, Columbia, SC 29208, USA; ^4^Health Promotion Research Center, University of Washington, 1107 NE 45th Street, Suite 200, Seattle, WA 98105, USA

Mobility is fundamental to everyday life and is critical to an understanding of the health and well-being of older populations. The construct of mobility focuses on the ability of individuals to meet the challenges of the environment given their capabilities associated with *movement within and between environments*. Mobility can also be characterized by its form including transferring from bed to a chair, walking and ambulation, engaging in activities associated with independence, work, leisure-time activities, driving a car, and using various forms of passenger transport (William Satariano, personal communication, April 24, 2011). It is a central tenet of successful aging [1] and healthy aging [2]. CDC's Healthy Aging Research Network defines healthy aging as “the development and maintenance of optimal physical, mental, and social well-being and function in older adults. It is most easily achieved when physical environments and communities are safe and support the adoption and maintenance of attitudes and behaviors known to promote health and well-being and when health services and community programs are used effectively to prevent or minimize the impact of acute and chronic disease on function” [2]. 

When we developed the call for papers for this special issue titled “Mobility and Aging: Transference to Transportation,” we viewed this as an opportunity to advance how we conceptualize mobility and recruit authors whose work would provide new insights into how mobility is framed from diverse perspectives and add critical thinking about community-based programs and policies to promote mobility in older populations. Another goal is to stimulate interest and dialogue among a broader community of researchers concerned with mobility. Overseeing the development of this special issue has enhanced our appreciation of the contributions being made to the field and expanded our thinking about options for individual, community, systems, and policy interventions to increase the impact of our individual work and the collective contributions as members of the CDC Healthy Aging Research Network (http://www.prc-han.org/). 

Although research literature exists for several forms of mobility, including use of transportation, driving, walking, use of assistive devices for ambulation (walkers, wheelchairs), and exercise, this research has largely focused on specific, individual forms of mobility without the benefit of a comprehensive unified framework for study. That is, research has typically focused on a single domain or form of mobility. This body of research tends to focus on a limited set of causal antecedents such as the role of the built environment and functional status as determinants of walking for leisure among older adults. Without a broadly-based unified framework to examine the spectrum of mobility disability across structural and personal antecedents, we will fail to fully identify and capture the factors needed to address the mobility needs of older populations. We may also potentially fail to see the commonalities across types of mobility and the potential for designing interventions, environments and policy to address mobility disability. For example, the influence of obesity and patterns of multiple chronic conditions across several types of mobility may not be apparent in research that addresses a single form of mobility. We, therefore, take this opportunity to provide new and critical ways of framing mobility from an integrated public health perspective. 

We examine several theoretical models that can guide a unified approach to mobility and aging research. We conclude with a call to action to move the science and the field forward. The significance of this field is apparent: the US Census 2000 counted 49.7 million people with some type of long-lasting condition or disability—whether due to age, injury, or birthrelated [3]. It is critical to ensure that all people have an opportunity to participate in valued activities. Mobility is more than an outcome or end point of the research; mobility restrictions have consequences for the health and well-being of older adults which often result in a cascade effect of continuing deterioration. 

The theoretical foundation that we propose can help guide an integrated mobility research agenda and is based on disablement models posed by the early work of Nagi [4] and later expanded by Verbrugge and Jette [5]. These disablement models distinguish between impairment (a body system disease or injury) and disability (a limitation in performing social roles and activities) while recognizing these activities are conducted in a setting that include a sociocultural and environmental context that influences the level of disability observed. More recently, the World Health Organization (WHO) proposed the International Classification of Functioning, Disability, and Health [6, ICF]. ICF was designed to provide a standard language and framework for describing health and health-related states. It includes a multipurpose classification of health (rather than just illness) and health-related domains. As in previous disablement models, ICF is a biopsychosocial model that integrates biological, individual, and social perspectives. Thus, the model recognizes that activities such as types of mobility are influenced by health conditions and contextual factors including the environmental and personal factors. Another feature of the framework important to understanding mobility is that it distinguishes between *performance *and* capacity*. *Performance *can be thought of as activities that are observable whereas *capacity* refers to a person's ability under optimal personal and situational contexts. In summary, ICF underscores the positive, or potential, abilities of the individual and provides a framework that stresses personal situational and environment influences. ICF framework can contribute to observational and intervention research on mobility in older populations and is consistent with the social-ecological framework that identifies personal, structural, and environmental factors that may influence mobility including the following.

- Modifying the environment through policy change to maximize mobility options.
- Providing appropriate assistive devices to enhance mobility.
- Improving the capacity or reserve through exercise and health-promoting strategies.
- Supporting assistance through informal support networks to address unmet mobility needs.
- Addressing beliefs, motivations, and perceptions about mobility limitations among individuals and families to help overcome “self-restricted” mobility limitations or effectively cope with the circumstances related to mobility restriction.

The challenge for future intervention research is to determine the most appropriate combination of intervention elements that would result in minimal restriction due to mobility limitations. Research focusing on both performance and capacity could help to determine the appropriate elements for the intervention.

As mentioned previously, we also need to think about the public health impact of the strategies we propose and how they can be brought to scale at a population level. The Social Ecological Model [7] and the Health Impact Pyramid [8] are useful in this regard. Similar to the ICF, within the Social Ecological Model the role of ecological factors which can influence individual behavior are noted. The model stresses the need to address interventions at multiple levels including the individual, interpersonal, institutional, community, and policy level. The Health Impact Pyramid provides an organizational structure for categorizing intervention strategies to promote health, or, in this case, mobility, with respect to their potential relative impact on the targeted health concern. The five tiers of the pyramid include socioeconomic factors, changing the context to make individuals' default decision healthy, long-lasting protective interventions, clinical intervention, and counseling and education. Beginning at the base of the pyramid with socioeconomic factors, interventions rise up from those that have the largest population impact and change the context to make healthy choices the default [8]. Depending on the causal antecedents and underlying risk factors (e.g., environmental, personal) an intervention on mobility restriction for an older population would have its greatest impact as close to the pyramid base as possible. Green and Kreuter [9] have further recommended that population intervention strategies should be blended depending on the complexity and etiology of the health concern. To use the example of walking among older individuals, an intervention to increase walking that focuses on increasing safe and desirable places to walk would fall in the category of a long-lasting protective intervention while an intervention that makes assistive devices readily available among disadvantaged older population with limited walking ability would be a socioeconomic intervention strategy. Similarly, a health education nutrition and exercise health promotion intervention to promote walking and lose weight would be a counseling and education intervention. While policy directed at promoting walking for health among older populations would have the greatest potential for reach and population level impact, we need to be cognizant of the fact that a *combined blended intervention strategy* is likely to be required to address situational and environmental barriers and opportunities [9]. 

We hope that this special issue is a call to action, helping to stimulate expanded and new research on mobility and aging. The diversity of the articles provide evidence that researchers recognize the range of mobility domains critical to independence of older populations and the broad array of structural, personal, and environmental factors effecting mobility. Additionally, the diversity of disciplines represented by the authors in this special issue attests to the collaborations that are essential if we are going to make progress. What is missing is a unified and comprehensive approach to mobility and aging research. We expect this editorial to stimulate discussion and challenge scholars and practitioners to think differently and act together to advance the field.

## References

[1] Depp CA, Jeste DV (2006). The definition and predictors of successful aging: a comprehensive literature review. *American Journal of Geriatric Psychiatry*.

[2] (2006). The Healthy Aging Research Network Writing Group. The Prevention Research Centers Healthy Aging Research Network. *Preventing Chronic Disease*.

[3] Waldrop J, Stern SM (2003). *Disability Status: 2000 [Census 2000 brief]*.

[4] Nagi S (1964). A study in the evaluation of disability and rehabilitation potential: concepts, methods, and procedures. *American Journal of Public Health*.

[5] Verbrugge L, Jette A (1994). The disablement process. *Social Science and Medicine*.

[6] (2002). World Health Organization. Toward a common language for functioning, disability and health. *ICF: The International Classification of Functioning, Disability and Health*.

[7] McLeroy KR, Bibeau D, Steckler A, Glanz K (1988). An ecological perspective on health promotion programs. *Health Education Quarterly*.

[8] Frieden T (2010). A framework for public health action: the health impact pyramid. *American Journal of Public Health*.

[9] Green L, Kreuter M (2010). Evidence hierarchies versus synergistic interventions. *American Journal of Public Health*.

